# Miniaturized Wearable System for Multimodal EEG/ECG/EMG Sensing and Real-Time Physiological Monitoring

**DOI:** 10.3390/mi17060697

**Published:** 2026-06-06

**Authors:** Yunxiang Zhang, Xueyang Meng, Chengbang Lu, Yingning He, Xiangyu Liang

**Affiliations:** 1 School of Physics and Optoelectronics, Xiangtan University, Xiangtan 411105, China; 2 Agricultural Genomics Institute at Shenzhen, Chinese Academy of Agricultural Sciences, Shenzhen 518120, China; 3 Institute of Bast Fiber Crops, Center of Southern Economic Crops, Chinese Academy of Agricultural Sciences, Changsha 410205, China; 4 State Key Laboratory of Molecular Engineering of Polymers, Fudan University, Shanghai 200438, China

**Keywords:** micro device, biosensing and diagnostics, multimodal, real-time monitoring, seizure detection

## Abstract

Real-time physiological state awareness is central to next-generation wearable computing, yet most existing electrophysiological signal acquisition platforms remain limited to single-modality sensing, high component cost, or bulky form factors that hinder everyday deployment. Here, we present a compact, low-cost wearable platform for simultaneous electroencephalography (EEG), electromyography (EMG), and electrocardiography (ECG) acquisition. The system integrates an analog front-end, a microcontroller, and a Bluetooth wireless link on a compact single-board platform (5.6 × 3.8 cm, approximately 12.8 g with the selected lithium-polymer battery installed), with an estimated bill-of-materials cost of 67.40 USD. Experimental validation across three healthy subjects, with the ECG channel additionally benchmarked against a commercial clinical-grade ambulatory ECG recorder, demonstrates that the platform captures ECG waveforms with recognizable P-QRS-T morphology under controlled recording conditions, supports reliable R-peak detection and heart rate estimation, records stable resting-state EEG spectral features, and distinguishes EMG activation from resting baseline in both time-domain amplitude and time-frequency structure. Leveraging the real-time wireless data link between the wearable hardware and a PC-hosted MATLAB environment, we further explore application-oriented signal processing scenarios. As an offline algorithm-pipeline compatibility demonstration, a CNN-based seizure detection pipeline is applied to the Bonn EEG benchmark for five-class epileptic state classification, achieving 86.60% mean classification accuracy. The proposed system offers a scalable and affordable foundation for wearable human-state-aware interaction, with potential applications in clinical monitoring, rehabilitation, and brain–computer interfaces.

## 1. Introduction

Wearable computing is progressively evolving from passive data logging toward systems capable of continuous physiological sensing and context-aware adaptive interaction [1,2]. By embedding sensing, processing, and communication into body-worn form factors, these systems open new possibilities for health monitoring, assistive technology, and human–computer interaction that operate unobtrusively in daily life [3,4]. Among the diverse landscape of physiological signals, electroencephalography (EEG), electromyography (EMG), and electrocardiography (ECG) constitute three complementary modalities that collectively span neural, muscular, and cardiovascular activity [5]. EEG provides a direct, non-invasive window into cortical dynamics, supporting applications in cognitive state classification, seizure detection, and brain–computer interfaces [6,7]. EMG captures the electrical manifestation of skeletal muscle activation, enabling gesture recognition, prosthetic control, and neuromuscular assessment [8,9]. ECG records the cardiac conduction cycle with high temporal fidelity, underpinning heart rate variability analysis, arrhythmia screening, and autonomic nervous system evaluation [10,11]. The concurrent acquisition of these three modalities within a single wearable system would therefore enable a substantially richer characterization of the wearer’s physiological state than any single modality alone.

Despite this promise, existing wearable biosensing platforms face several persistent challenges. First, many systems are designed around a single modality, necessitating separate devices for each signal type and thereby increasing system complexity, power consumption, and user burden [12,13]. Second, research-grade multi-channel EEG systems—while offering high signal fidelity—typically rely on expensive analog front-end integrated circuits and laboratory-oriented form factors that are impractical for mobile or out-of-clinic use [14,15]. Third, platforms that do support multimodal acquisition often lack real-time wireless streaming capability, confining data analysis to post-session offline processing and precluding closed-loop adaptive interaction [16,17]. Collectively, these constraints limit the translation of multimodal physiological sensing from controlled laboratory settings to the everyday wearable scenarios where its potential impact is greatest.

Recent advances in low-power analog front-end (AFE) technology, embedded microcontrollers, and short-range wireless protocols have begun to narrow this gap [18,19]. Integrated AFE devices now offer multi-channel, simultaneous sampling with programmable gain and built-in bias generation, substantially reducing external component count [20]. Ultra-low-power microcontroller units (MCUs) provide sufficient computational throughput for real-time signal conditioning while maintaining milliwatt-level energy budgets compatible with battery-powered wearable operation [21]. Meanwhile, Bluetooth Classic and Bluetooth Low Energy protocols offer standardized, low-latency data links to smartphones and personal computers with minimal additional silicon area [22]. The convergence of these building blocks creates a timely opportunity to realize compact, affordable, and multimodal wearable sensing platforms.

A representative scenario in which simultaneous EEG, ECG, and EMG acquisition is essential is seizure monitoring, where EEG serves as the primary modality and ECG and EMG act as complementary channels. While epileptiform discharges on EEG remain the principal diagnostic criterion, peri-ictal heart rate changes (ictal tachycardia, post-ictal bradycardia) and tonic–clonic activity on EMG provide concurrent physiological signatures that can be used to cross-validate suspected events, suppressing false positives caused by chewing artifacts, head motion, or transient electrode loosening. Such a primary-with-auxiliary cross-modal verification scheme is unattainable with any single-modality system and is one of the core motivations for integrating all three modalities into the present platform.

**Figure 1:**
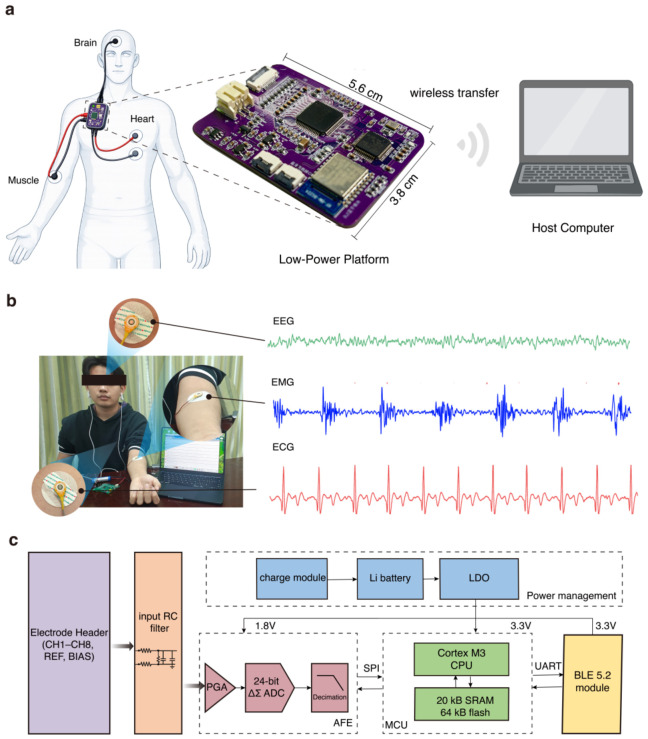
Overview of the miniaturized wearable multimodal biosensing platform. (**a**) Body-centric electrode placement with the assembled PCB (5.6 × 3.8 cm) and wireless data link to a host computer. (**b**) Photographs of the experimental setup showing EEG acquisition via scalp-mounted electrodes, EMG recording from the forearm, and ECG capture from the chest, alongside representative waveform previews for each modality. (**c**) System block diagram of the signal chain from the electrode header through input RC filtering, programmable gain amplification, digital decimation, and high-resolution ADC to the microcontroller and wireless communication module, with integrated power management for charging, battery supply, and voltage regulation.

In this work, we present the design, implementation, and experimental validation of such a platform. The system integrates EEG, EMG, and ECG acquisition channels into a unified hardware architecture consolidated on a single printed circuit board. As illustrated in Figure 1, the proposed platform combines body-level deployment, practical multimodal recording, and an integrated system signal chain within a compact wearable format. Specifically, Figure 1a shows the overall wearable configuration, in which the miniaturized PCB is connected to body-surface electrodes and wirelessly transmits physiological data to an external host computer. Figure 1b presents photographs of the actual experimental setup for multimodal acquisition, including scalp EEG recording, forearm EMG measurement, and chest ECG monitoring, together with representative waveform previews from each modality, thereby demonstrating the practical operation of the system in real recording scenarios. Figure 1c summarizes the internal hardware pathway, where an ADS1299 analog front-end performs multi-channel signal conditioning, an STM32F103C8T6 microcontroller handles digitization, real-time control, and data packetization, and an EFR32BG22 BLE 5.2 module provides continuous wireless streaming to an external host. The overall bill-of-material cost is maintained below 70 USD, making the platform accessible for resource-constrained research and educational settings. We validate the system through a series of experiments spanning all three modalities. ECG recordings obtained during prolonged resting-state monitoring confirm well-defined cardiac waveform morphology, accurate R-peak detection, and stable heart rate statistics across multiple recording segments. Multi-channel resting-state EEG analysis reveals characteristic spectral distributions across canonical frequency bands with adequate signal quality for clinical interpretation. EMG measurements under resting and voluntary contraction conditions demonstrate clear activation discrimination in both time-domain and time–frequency representations. Finally, building upon the real-time wireless data pathway established between the wearable board and a PC-hosted MATLAB environment, we explore application-oriented processing scenarios. As a representative offline algorithm demonstration, a CNN-based seizure detection pipeline is constructed and validated on the Bonn EEG benchmark dataset [23] for five-class epileptic state classification, achieving 86.60% mean classification accuracy and illustrating the compatibility of the MATLAB-hosted analysis workflow with downstream intelligent signal-processing pipelines.

## 2. Materials and Methods

### 2.1. Hardware Implementation

The analog front-end was built around an ADS1299 (Texas Instruments, Dallas, TX, USA), configured for eight-channel simultaneous differential sampling at 250 Hz with a programmable gain of ×24 and 24-bit resolution. The integrated bias drive circuit provided active common-mode rejection. Digitized data were read via SPI by an STM32F103C8T6 microcontroller (STMicroelectronics, Geneva, Switzerland), packetized, and forwarded over UART to an EFR32BG22-based BLE 5.2 module (RF-BM-BG22A3, RF-star, Shenzhen, China) interfaced via UART. The power subsystem used LP5907 and TLV700-series low-dropout regulators (Texas Instruments, Dallas, TX, USA) to generate separate analog and digital supply rails from a 3.7 V lithium-polymer battery, with dedicated LC filtering on the AFE supply. All components were integrated on a 5.6 × 3.8 cm two-layer PCB.

### 2.2. Electrode Configuration

Scalp EEG was recorded using a four-channel bipolar montage (F7–T3, T3–T5, Fp1–F7, and P3–O1) with wet Ag/AgCl electrodes mounted on an elastic headband. Surface EMG was acquired from biceps brachii of the dominant upper arm using a bipolar electrode pair with approximately 30 mm inter-electrode distance. Single-lead ECG was captured via adhesive gel electrodes placed at the left precordial region. Electrode impedance was maintained below 100 kΩ for all channels prior to recording.

### 2.3. Participants and Ethics

Three healthy male volunteers aged 24, 25, and 27 years participated in the study. All participants provided written informed consent. This study was conducted in accordance with the Declaration of Helsinki, and the protocol was approved by the Ethics Committee of Shenzhen Guangming District People’s Hospital (Ethics Approval No. LL-KT-2025144; date of approval: 15 December 2025).

### 2.4. Data Acquisition Protocol

For ECG validation, a continuous resting-state recording of 50 s was obtained with the subject seated in a relaxed posture. Resting-state EEG was recorded for 5 min with eyes closed in a quiet, dimly lit room. EMG was recorded during alternating blocks of ~1 s voluntary isometric elbow flexion and ~1 s rest (cycle duration ~2 s), repeated for 20 cycles. Across the three subjects, EEG, EMG, and ECG were additionally acquired in a simultaneous tri-modal configuration. To benchmark the platform’s ECG channel against clinical equipment, an additional session was recorded with our device connected in parallel to a commercial clinical-grade ambulatory ECG/blood-pressure recorder (CB-2304-A, BIOX Instruments Co., Ltd., Wuxi, China) on the same subject, with both systems recording the same lead simultaneously to allow direct comparison.

### 2.5. Signal Processing

All signal processing was performed in MATLAB R2023a (MathWorks, Natick, MA, USA). Raw signals were band-pass-filtered (fourth-order Butterworth; EEG: 0.5–50 Hz; EMG: 5–50 Hz; and ECG: 0.5–50 Hz) and notch-filtered at 50 Hz and 60 Hz to suppress power-line interference. ECG R-peaks were detected using MATLAB’s findpeaks function with automatic polarity correction, from which R–R intervals and instantaneous heart rate were derived. EEG power spectral density was estimated by Welch’s method (window length 1 s, 50% overlap, Hamming window, and NFFT = 256), and band power was computed by integrating the PSD over delta (0.5–4 Hz), theta (4–8 Hz), alpha (8–13 Hz), and beta (13–30 Hz) ranges. EMG root-mean-square envelopes were calculated with a sliding window of 500 ms and a step size of 100 ms. Time–frequency representations were generated via short-time Fourier transform (STFT; 500 ms Hamming window, 80% overlap, and NFFT = 256).

### 2.6. Seizure Detection Network

A CNN-based epileptic state classification pipeline was implemented in MATLAB to demonstrate application-level compatibility. The Bonn EEG benchmark dataset [23] was used, comprising 500 single-channel EEG segments equally distributed across the following five classes: Z (healthy, eyes open), O (healthy, eyes closed), N (interictal, hippocampal formation), F (interictal, epileptogenic zone), and S (seizure activity). Each segment contains 4097 samples recorded at 173.61 Hz. Raw signals were transformed into time–frequency representations via STFT (window length 256 samples, 50% overlap, and NFFT = 512), and the resulting spectrograms were used as image inputs to the CNN. The network comprised three convolutional blocks (32, 64, and 128 filters; 3 × 3 kernels; each followed by batch normalization, ReLU activation, and 2 × 2 max-pooling), a fully connected layer (128 units, ReLU, dropout rate 0.3), and a five-class softmax output. Training used the Adam optimizer (learning rate 1 × 10^−4^, batch size 16) for 30 epochs. Model performance was evaluated using stratified five-fold cross-validation, reporting per-fold accuracy, mean accuracy, and standard deviation, alongside an overall confusion matrix aggregated across all folds.

### 2.7. Statistical Analysis

Heart rate values across recording segments were compared descriptively using mean ± standard deviation. All signal processing and analyses were performed in MATLAB.

## 3. Results

### 3.1. System Architecture and Hardware Implementation

The platform adopts modular architecture comprising the following four functional stages: sensing interface, analog front-end (AFE), digital processing unit, and wireless communication module. At the sensing interface, body-surface electrodes are placed at three anatomical sites to capture distinct electrophysiological modalities. A headband-mounted electrode array acquires multi-channel EEG from the scalp, a forearm band records EMG from the biceps brachii, and adhesive chest electrodes capture single-lead ECG. All electrode channels are routed through a shared connector to the main board, enabling modular reconfiguration without hardware modification.

The AFE stage is built around an ADS1299 (Texas Instruments) integrated circuit, which provides eight simultaneously sampled differential input channels, a programmable gain amplifier (PGA) with gains up to ×24, and an integrated bias drive circuit for common-mode rejection. The on-chip 24-bit delta-sigma ADC operates at a configurable sampling rate of 250 Hz, digitizing microvolt-level EEG and millivolt-level ECG/EMG signals through a single unified data path. This integration eliminates the need for discrete instrumentation amplifiers, anti-aliasing filters, and bias resistor networks that would otherwise dominate board area and cost. The digitized data are transferred via SPI to an STM32F103C8T6 microcontroller, which serves as the central coordinator. The MCU performs real-time tasks including data packetization and peripheral management, operating at 72 MHz. Its 64 KB flash and 20 KB SRAM are sufficient to buffer multiple sample frames before transmission, accommodating transient Bluetooth throughput fluctuations without data loss. The wireless link is provided by an EFR32BG22-based BLE 5.2 module (RF-BM-BG22A3, RF-star) interfaced to the MCU via UART. In the current implementation, Bluetooth Low Energy (BLE 5.2) is used to stream packetized data to a host PC where a MATLAB-based receiver reconstructs the multi-channel time series in real time. This wired-free data pathway enables continuous monitoring without constraining the subject’s movement and provides a direct interface for real-time signal visualization and algorithm development within the MATLAB environment. The power subsystem accepts a 3.7 V lithium-polymer battery input and generates regulated analog and digital supply rails through low-dropout regulators, with dedicated filtering to minimize supply-coupled noise on the AFE. The complete system is consolidated on a 5.6 × 3.8 cm two-layer PCB. The assembled wearable device weighs approximately 12.8 g with the selected lithium battery installed, excluding lead wires, front-end electrodes, and the elastic headband. The total bill-of-material cost, covering all active and passive components, and two-layer PCB fabrication, the selected lithium battery, and lead wires is approximately 67.40 USD at single-unit prototype quantity; front-end electrodes are excluded, as the system is compatible with various commercially available electrode types. A detailed bill of materials is provided in Table S1, Supporting Information.

### 3.2. ECG Signal Acquisition and Cardiac Rhythm Validation

To assess the platform’s ECG acquisition capability, a continuous single-lead recording was obtained from one healthy subject during resting-state monitoring.

**Figure 2:**
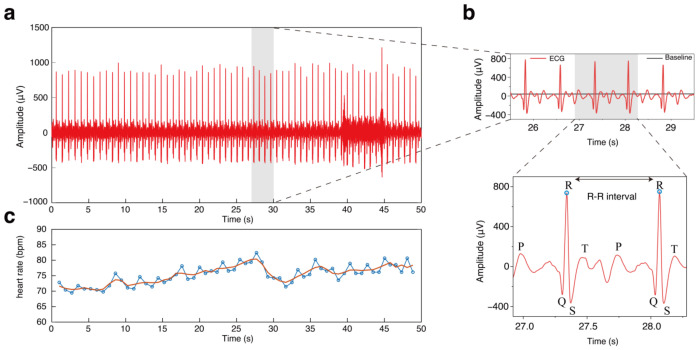
ECG signal validation of the proposed wearable platform. (**a**) Continuous single-lead ECG recording over 50 s. (**b**) Progressive zoom-in: upper panel shows a 3 s segment with clearly resolved cardiac cycles; lower panel further magnifies two consecutive heartbeats with annotated P, Q, R, S, and T wave components and R–R interval. (**c**) Beat-to-beat instantaneous heart rate derived from R–R intervals, with a smoothed trend line indicating stable cardiac rhythm throughout the recording. The blue line with circular markers represents the beat-to-beat instantaneous heart rate, and the red line represents the smoothed trend line indicating stable cardiac rhythm throughout the recording.

Figure 2a presents the full 50 s ECG trace with automatically detected R-peaks overlaid as markers. The waveform exhibits clearly delineated cardiac cycles throughout the recording, with no visible saturation or baseline drift indicative of amplifier overload or electrode degradation. A progressive zoom-in (Figure 2b) first reveals several consecutive heartbeats at a 3 s scale, then further magnifies two individual cardiac cycles with annotated P, Q, R, S, and T wave components and a measured R–R interval, confirming that the system’s bandwidth and dynamic range preserve the morphological features required for clinical interpretation.

Beat-to-beat instantaneous heart rate derived from R–R intervals is shown in Figure 2c. The heart rate fluctuates within a range of approximately 69–82 bpm over the 50 s recording, with a smoothed trend line indicating a gradual and physiologically expected drift. The absence of abrupt discontinuities or outliers confirms reliable R-peak detection and stable signal acquisition throughout the session.

These results demonstrate that the platform captures ECG signals of sufficient quality for continuous heart rate monitoring, R-peak-based timing analysis, and heart rate variability studies in ambulatory settings.

To assess the reliability of the platform’s ECG channel relative to clinical monitoring equipment, our device was connected in parallel with a commercial clinical-grade ambulatory ECG/blood-pressure recorder (CB-2304-A, BIOX Instruments Co., Ltd., Wuxi, China) on the same subject for synchronous recording. Representative synchronized waveforms are shown in Figure S2, and quantitative agreement is summarized in Table S2. The two systems showed consistent ECG waveform morphology and closely aligned R-peak timing, with differences within approximately 0.5 bpm for mean heart rate, 5 ms for mean R–R interval, and 2 ms for QRS duration. These results indicate that the platform’s ECG output is consistent with the key timing parameters obtained from the clinical-grade reference device under controlled recording conditions.

### 3.3. EMG Signal Characterization Under Resting and Active Conditions

**Figure 3:**
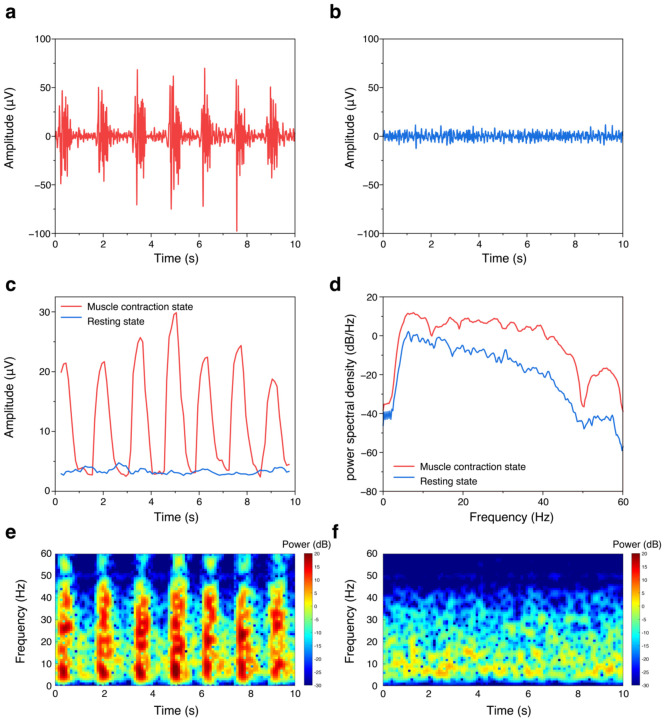
Comparative analysis of EMG signals under active contraction (red) and resting (blue) conditions. (**a**,**b**) Raw EMG waveforms showing high-amplitude burst activity during contraction versus low-amplitude baseline at rest. (**c**) Overlaid root-mean-square (RMS) envelopes of both states. (**d**) Power spectral density comparison, revealing broadband elevation during muscle activation. (**e**,**f**) Time–frequency spectrograms showing periodic broadband energy bursts during contraction versus a uniformly low-energy background at rest.

Surface EMG was recorded from the biceps brachii of the dominant upper arm during the following two contrasting conditions: voluntary isometric contraction of the elbow flexors and relaxed resting baseline. Figure 3 presents a multi-level comparison organized in four analytical domains.

In the time domain (Figure 3a,b), the contraction-state signal displays high-amplitude, dense burst activity with peak excursions reaching approximately ±100 µV, whereas the resting-state trace remains near the noise floor with amplitudes below ±20 µV. This amplitude contrast provides immediate visual confirmation of the system’s dynamic range and its ability to resolve voluntary muscle activation from background noise. The root-mean-square (RMS) envelope (Figure 3c) overlays both conditions in a single panel, directly visualizing the energy contrast. During contraction, the RMS curve reveals rhythmic modulations with a period of approximately 2 s, corresponding to the subject’s cyclic contraction-and-relaxation pattern. Peak RMS values reach approximately 25–30 µV, representing a roughly six-fold increase over the resting baseline RMS of 3–5 µV. This metric provides a computationally lightweight feature suitable for real-time activation detection in embedded or streaming applications.

Power spectral density analysis (Figure 3d) provides a frequency-domain perspective on the two conditions. The contraction-state spectrum exhibits elevated power across approximately 5–50 Hz relative to the resting baseline, with a difference of approximately 8–18 dB/Hz across the primary EMG frequency range. The separation is most pronounced in the 15–40 Hz band (~10–18 dB/Hz) and increases further near 45–50 Hz (~20–25 dB/Hz). The resting-state spectrum remains below −40 dB/Hz across the full bandwidth. This distinct spectral separation further confirms the system’s ability to capture physiologically meaningful EMG features. Time–frequency spectrograms (Figure 3e,f) offer a fourth, complementary perspective. The contraction-state spectrogram shows broadband energy elevation spanning approximately 5–60 Hz, with power concentrated in periodic bursts that align temporally with the RMS peaks. In contrast, the resting-state spectrogram presents a uniformly low-energy background with no structured activation patterns. This joint time–frequency representation resolves ambiguities that neither time-domain amplitude nor RMS alone can address: transient motion artifacts may produce comparable RMS peaks, but their spectral signatures differ markedly from physiological muscle activation, enabling more reliable event discrimination.

### 3.4. Resting-State EEG Spectral Characterization

Multi-channel EEG was recorded from one subject in a relaxed, eyes-closed resting state using a four-channel bipolar montage (F7–T3, T3–T5, Fp1–F7, and P3–O1) covering temporal and parieto-occipital regions.

**Figure 4:**
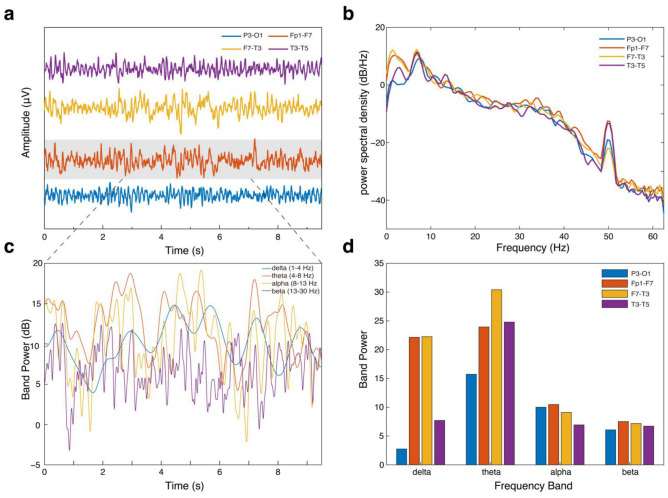
Resting-state EEG spectral characterization. (**a**) Raw multi-channel EEG waveforms from four bipolar derivations (P3–O1, Fp1–F7, F7–T3, T3–T5), with an inset showing an enlarged segment of the Fp1–F7 channel. (**b**) Overlaid power spectral density (PSD) estimates across channels. (**c**) Time-resolved band-power curves showing dynamic spectral fluctuations over the recording period. (**d**) Band-power distribution for delta, theta, alpha, and beta frequency bands by channel.

Figure 4a displays a 10 s segment of the raw multi-channel EEG, with an inset showing an enlarged view of the Fp1–F7 channel. All channels exhibit continuous oscillatory activity with peak-to-peak amplitudes in the range of 40–80 µV, consistent with normal scalp-recorded EEG. The temporal channels (F7–T3, T3–T5) show relatively higher amplitude fluctuations compared to the parieto-occipital derivation (P3–O1), reflecting the known regional variation in cortical source geometry and electrode proximity.

Power spectral density (PSD) estimates computed over the full recording epoch (Figure 4b) reveal the expected 1/f spectral slope with superimposed band-specific peaks. All channels exhibit a spectral maximum in the 5–8 Hz (theta) range, with peak power densities of approximately 8–12 dB/Hz for the temporal channels. The P3–O1 channel shows notably lower power in the delta and theta bands but maintains a moderate alpha-band peak, consistent with posterior alpha rhythm during eyes-closed rest. A pronounced attenuation of 20–30 dB is observed above 40 Hz across all channels, with a sharp notch near 50 Hz corresponding to power-line interference rejection.

Time-resolved band-power curves (Figure 4c) track the dynamic fluctuation of each spectral component over the recording. Theta power fluctuates between approximately 15 and 20 dB with quasi-periodic modulations on the order of 1–2 s. Delta and alpha bands oscillate in the 10–15 dB range, while beta power remains the most stable at 5–10 dB. These spontaneous modulations reflect cortical state transitions characteristic of relaxed wakefulness, and their temporal consistency across the recording duration further supports the system’s low-noise performance over sustained monitoring.

Band-power quantification (Figure 4d) further decomposes the spectral content into canonical frequency bands. Theta power dominates across most channels, with F7–T3 exhibiting the highest value (~31 dB) followed by T3–T5 (~25 dB) and Fp1–F7 (~24 dB). Delta power is prominent in the temporal channels (Fp1–F7 and F7–T3, both ~22 dB) but markedly lower in P3–O1 (~3 dB). Alpha power is relatively uniform across channels (7–11 dB), while beta power remains the lowest (6–8 dB). The P3–O1 channel shows a distinctly higher alpha-to-delta ratio compared to the temporal derivations, consistent with the known spatial distribution of posterior alpha generators versus mesial temporal slow-wave sources. The spectral characterization presented above confirms that the platform captures EEG signals with sufficient frequency resolution and signal-to-noise ratio to resolve clinically meaningful spectral features across the delta through beta range. This capability is a prerequisite for spectrogram-based classification approaches, in which time–frequency representations serve as input features for pattern recognition algorithms. To further evaluate the platform’s compatibility with such downstream intelligent processing pipelines, we next apply the same STFT-based feature extraction strategy to a standardized EEG benchmark dataset for automated epileptic state classification. Representative simultaneous tri-modal recordings from the two additional subjects are provided in Figure S1 of the Supporting Information. These recordings were obtained in a parallel acquisition mode using the same wearable platform, confirming that the system can synchronously acquire ECG, EEG, and EMG signals under the tested conditions.

### 3.5. CNN-Based Seizure Detection as an Application Demonstration

Having established a real-time wireless data pathway from the wearable board to a PC-hosted MATLAB environment, we sought to demonstrate the platform’s suitability for application-level intelligent processing. EEG-based epileptic seizure classification was selected as a representative task due to its clinical significance and its demand for robust time–frequency pattern recognition.

**Figure 5:**
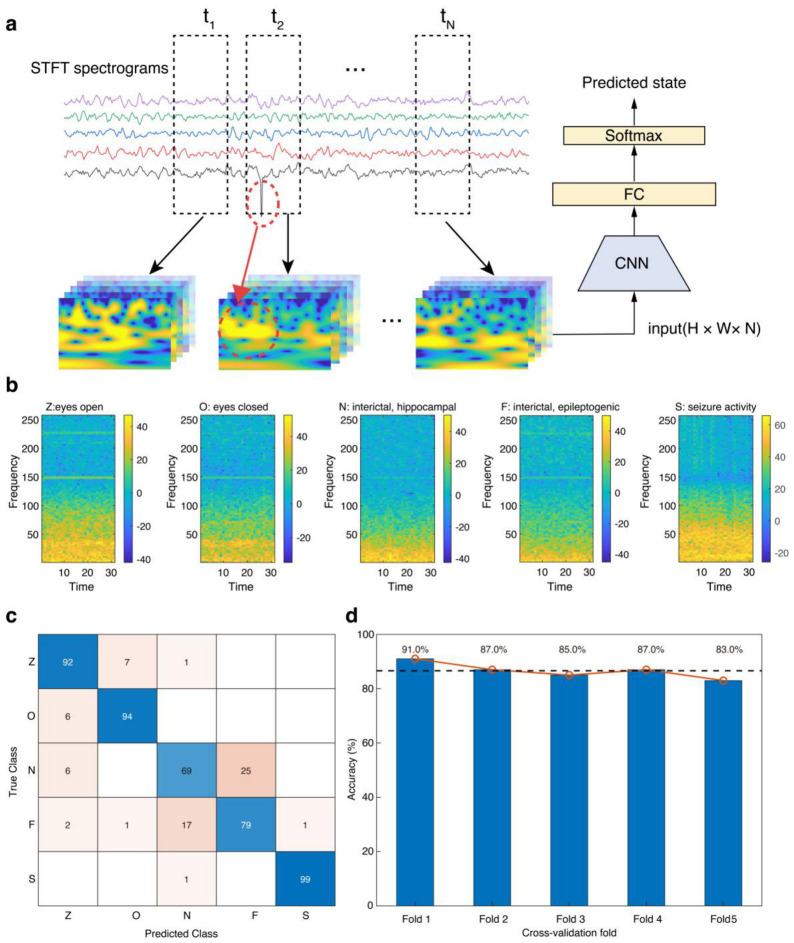
CNN-based five-class epileptic state classification on the Bonn EEG dataset. (**a**) Schematic of the processing pipeline: EEG signals are segmented and converted to time–frequency representations via STFT, then fed into a CNN comprising convolutional, fully connected, and softmax layers. (**b**) Example spectrograms for each class: Z (healthy, eyes open), O (healthy, eyes closed), N (interictal, hippocampal), F (interictal, epileptogenic zone), and S (ictal), warmer colors indicate higher spectral power and cooler colors indicate lower spectral power. (**c**) Overall confusion matrix aggregated across five folds, blue color intensity represents the number of correctly classified samples, whereas red color intensity indicates misclassified samples. (**d**) Per-fold classification accuracy with a mean of 86.60% ± 2.97%.

A lightweight CNN was implemented in MATLAB and evaluated on the Bonn EEG dataset [23], a widely used benchmark comprising 500 single-channel EEG segments across the following five classes: Z (healthy, eyes open), O (healthy, eyes closed), N (interictal, hippocampal), F (interictal, epileptogenic zone), and S (seizure activity). Each segment contains 4097 samples at 173.61 Hz. Raw signals were transformed into time–frequency image representations via short-time Fourier transform (window length 256 samples, 50% overlap, and NFFT = 512), and the resulting spectrograms were used as CNN inputs (Figure 5a).

The CNN architecture consists of three convolutional blocks (32, 64, and 128 filters respectively; 3 × 3 kernels; batch normalization; ReLU activation; 2 × 2 max-pooling), followed by a fully connected layer (128 units, dropout rate 0.3) and a five-class softmax output. Training used the Adam optimizer (learning rate 1 × 10^−4^, batch size 16) for 30 epochs, with a stratified five-fold cross-validation protocol to ensure unbiased performance estimation. The overall confusion matrix aggregated across all five folds is shown in Figure 5c. The model achieves a mean classification accuracy of 86.60% ± 2.97% across the five folds (Figure 5d), with per-fold accuracies ranging from 83.0% to 91.0%. Per-class analysis reveals that the S (seizure activity) and O (healthy, eyes closed) classes are classified with the highest accuracy, while the N and F interictal classes exhibit moderate confusion due to their spectral similarity.

The Bonn-dataset experiment described above constitutes an offline algorithm-pipeline demonstration using an open-source EEG benchmark dataset. It demonstrates that STFT-based feature extraction and CNN-based epileptic state classification can be implemented within the MATLAB-hosted analysis environment associated with the wearable platform. However, because the classification was performed on the Bonn dataset rather than on epileptic EEG acquired by the proposed wearable device, this result should not be interpreted as end-to-end clinical validation of the platform for seizure detection. Prospective validation using platform-acquired EEG from epilepsy patients, including synchronized ictal and interictal recordings, will be required in future studies.

## 4. Discussion

### 4.1. Signal Acquisition Performance and Application Scope

The experimental results validate the platform’s capability to acquire clinically interpretable signals across all three modalities within a compact, low-cost form factor. For ECG, the clearly resolved P-QRS-T morphology and stable beat-to-beat heart rate tracking confirm suitability for ambulatory heart rate monitoring and heart rate variability research, though the single-lead configuration does not support multi-lead diagnostic applications such as ischemic event localization. Extending to a two- or three-lead configuration is straightforward given the available AFE input channels.

For EEG, the four-channel temporal–parieto-occipital montage yields spectral characteristics consistent with established neurophysiological expectations, including dominant theta power in temporal derivations and posterior alpha rhythm in the P3–O1 channel. This coverage supports state-level classification tasks—drowsiness detection, seizure onset screening, and cognitive load estimation—but is insufficient for high-resolution source localization, which typically requires 32–128 channels. The modular architecture permits channel expansion through AFE cascading, at the expense of increased board area and power consumption.

For EMG, the clear separation between contraction and resting states in time-domain amplitude, RMS energy, spectral content, and time–frequency structure validates the platform for binary activation detection and fatigue monitoring. Graded force estimation or multi-gesture classification would require higher spatial electrode density and more advanced decomposition algorithms.

Compared with existing commercial and academic platforms, the proposed system offers a distinctive combination of simultaneous three-modality acquisition, 24-bit signal resolution, real-time wireless streaming, and a bill-of-material cost below 70 USD—a feature set not jointly available in current alternatives [15,24,25].

### 4.2. Real-Time Data Pathway and Intelligent Processing

A key contribution of this work is the established real-time wireless data link from the wearable hardware to a MATLAB host. The CNN-based five-class epileptic state classification on the Bonn EEG benchmark dataset confirms that the data format and throughput are compatible with standard machine learning workflows. Translating this capability into a closed-loop wearable application requires addressing the host-side computational bottleneck. The following two migration paths are foreseeable: (i) porting the classifier to a smartphone via optimized inference engines (e.g., TensorFlow Lite, ONNX Runtime), leveraging the existing BLE link; and (ii) on-device inference on the STM32F103C8T6 or an auxiliary co-processor, which would necessitate model compression to fit within the MCU’s computational budget.

### 4.3. Limitations and Future Directions

Several limitations of this study should be acknowledged. First, although the experimental validation has been extended to three subjects, the cohort remains small; larger-scale, multi-site, and multi-population testing is still required to establish broad generalizability. Second, the platform’s ECG channel has been benchmarked against a commercial clinical-grade ambulatory ECG/blood-pressure recorder through simultaneous co-recording (Figure S2 and Table S2); a more comprehensive benchmark covering all three modalities against gold-standard research instruments remains a direction for future work. Third, the CNN epileptic state classification was performed on the Bonn EEG benchmark dataset rather than on data acquired from the platform itself; while this validates pipeline compatibility, it does not directly characterize end-to-end classification performance with platform-acquired signals. Fourth, the current BLE 5.2 link provides adequate throughput for the present channel count but may become a bottleneck upon expansion to 16 or more channels, at which point migration to Wi-Fi would be advisable. Finally, the wet gel electrodes used in the current prototype, while beneficial for signal quality, limit long-term wearability; integration with dry or textile electrode technologies is an important direction for sustained ambulatory deployment.

## 5. Conclusions

We have developed a single-board wearable platform that unifies EEG, EMG, and ECG acquisition with real-time Bluetooth streaming to a PC-hosted analysis environment, at a bill-of-material cost of 67.4 USD. Across three healthy subjects and three signal modalities, the system produces recordings whose spectral content, waveform morphology, and activation contrasts are consistent with expected physiological patterns under controlled recording conditions. The ECG channel was additionally benchmarked against a commercial clinical-grade ambulatory ECG/blood-pressure recorder, showing close agreement in heart rate and ECG timing parameters. These results support the platform’s viability as a research-oriented multimodal sensing prototype, while comprehensive clinical-grade validation across larger and more diverse populations remains a necessary direction for future work. The demonstrated real-time data link to MATLAB, together with the CNN-based epileptic state classification benchmark on the open-source Bonn EEG dataset, establishes a proof-of-concept pathway from signal acquisition to offline intelligent signal-processing workflows. Future work will focus on expanding channel count, integrating dry electrode technology for improved long-term wearability, conducting multi-site clinical validation with gold-standard co-recording, and migrating classification algorithms to edge devices to realize fully autonomous closed-loop physiological state-aware interaction.

## Data Availability

The Bonn EEG benchmark dataset is publicly available at https://neurophysicsbonn.de/downloads (accessed on 2 June 2026). Platform hardware design files and signal processing scripts are available from the corresponding author upon reasonable request.

## Supplementary Materials

The following supporting information can be downloaded at https://www.mdpi.com/article/10.3390/mi17060697/s1, Figure S1: Representative simultaneous tri-modal recordings from two additional healthy subjects. (a,b) ECG, EEG, and EMG signals acquired in parallel from subjects S2 and S3, respectively, using the proposed wearable platform. Figure S2: ECG validation against a clinical-grade ambulatory ECG recorder. Representative synchronized ECG waveforms show consistent R-peak alignment between the two systems. Table S1: Bill of materials of the proposed wearable platform at single-unit prototype quantity. Table S2: Quantitative agreement between the proposed platform and the clinical-grade ambulatory ECG recorder on synchronously recorded ECG.

## Abbreviations

The following abbreviations are used in this manuscript:

EEG	Electroencephalography
EMG	Electromyography
ECG	Electrocardiography
AFE	Analog Front-End
ADC	Analog-to-Digital Converter
MCU	Microcontroller Unit
PGA	Programmable Gain Amplifier
SPI	Serial Peripheral Interface
UART	Universal Asynchronous Receiver-Transmitter
BLE	Bluetooth Low Energy
PCB	Printed Circuit Board
SRAM	Static Random-Access Memory
LDO	Low-Dropout Regulator
PSD	Power Spectral Density
RMS	Root Mean Square
STFT	Short-Time Fourier Transform
CNN	Convolutional Neural Network
BCI	Brain–Computer Interface
HRV	Heart Rate Variability
PPG	Photoplethysmography
SNR	Signal-to-Noise Ratio
BOMs	Bill of Materials

## References

[1] Patel S., Park H., Bonato P., Chan L., Rodgers M. (2012). A Review of Wearable Sensors and Systems with Application in Rehabilitation. J. NeuroEng. Rehabil..

[2] Ometov A., Shubina V., Klus L., Skibińska J., Saafi S., Pascacio P., Flueratoru L., Gaibor D.Q., Chukhno N., Chukhno O. (2021). A Survey on Wearable Technology: History, State-of-the-Art and Current Challenges. Comput. Netw..

[3] Heikenfeld J., Jajack A., Rogers J., Gutruf P., Tian L., Pan T., Li R., Khine M., Kim J., Wang J. (2018). Wearable Sensors: Modalities, Challenges, and Prospects. Lab Chip.

[4] Pantelopoulos A., Bourbakis N.G. (2010). A Survey on Wearable Sensor-Based Systems for Health Monitoring and Prognosis. IEEE Trans. Syst. Man. Cybern. Part C (Appl. Rev.).

[5] Chi Y.M., Jung T.-P., Cauwenberghs G. (2010). Dry-Contact and Noncontact Biopotential Electrodes: Methodological Review. IEEE Rev. Biomed. Eng..

[6] Lotte F., Bougrain L., Cichocki A., Clerc M., Congedo M., Rakotomamonjy A., Yger F. (2018). A Review of Classification Algorithms for EEG-Based Brain–Computer Interfaces: A 10 Year Update. J. Neural Eng..

[7] Craik A., He Y., Contreras-Vidal J.L. (2019). Deep Learning for Electroencephalogram (EEG) Classification Tasks: A Review. J. Neural Eng..

[8] Bi L., Feleke A.G., Guan C. (2019). A Review on EMG-Based Motor Intention Prediction of Continuous Human Upper Limb Motion for Human-Robot Collaboration. Biomed. Signal Process. Control..

[9] Phinyomark A., Scheme E. (2018). EMG Pattern Recognition in the Era of Big Data and Deep Learning. Big Data Cogn. Comput..

[10] Kaplan Berkaya S., Uysal A.K., Sora Gunal E., Ergin S., Gunal S., Gulmezoglu M.B. (2018). A Survey on ECG Analysis. Biomed. Signal Process. Control..

[11] Shaffer F., Ginsberg J.P. (2017). An Overview of Heart Rate Variability Metrics and Norms. Front. Public Health.

[12] Casson A.J. (2019). Wearable EEG and Beyond. Biomed. Eng. Lett..

[13] Majumder S., Mondal T., Deen M.J. (2017). Wearable Sensors for Remote Health Monitoring. Sensors.

[14] Ratti E., Waninger S., Berka C., Ruffini G., Verma A. (2017). Comparison of Medical and Consumer Wireless EEG Systems for Use in Clinical Trials. Front. Hum. Neurosci..

[15] Mihajlović V., Grundlehner B., Vullers R., Penders J. (2015). Wearable, Wireless EEG Solutions in Daily Life Applications: What Are We Missing?. IEEE J. Biomed. Health Inform..

[16] Xu J., Mitra S., Van Hoof C., Yazicioglu R.F., Makinwa K.A.A. (2017). Active Electrodes for Wearable EEG Acquisition: Review and Electronics Design Methodology. IEEE Rev. Biomed. Eng..

[17] Kim D.-H., Ghaffari R., Lu N., Rogers J.A. (2012). Flexible and Stretchable Electronics for Biointegrated Devices. Annu. Rev. Biomed. Eng..

[18] Yazicioglu R.F., Kim S., Torfs T., Kim H., Van Hoof C. (2011). A 30 μ W Analog Signal Processor ASIC for Portable Biopotential Signal Monitoring. IEEE J. Solid-State Circuits.

[19] Ha U., Lee Y., Kim H., Roh T., Bae J., Kim C., Yoo H.-J. (2015). A Wearable EEG-HEG-HRV Multimodal System with Simultaneous Monitoring of tES for Mental Health Management. IEEE Trans. Biomed. Circuits Syst..

[20] Rashid U., Niazi I.K., Signal N., Taylor D. (2018). An EEG Experimental Study Evaluating the Performance of Texas Instruments ADS1299. Sensors.

[21] Barras M., Booth L., Bateson A.D., Asghar A.U.R., Zeinali M., Mehmood A. (2026). Toward Mobile Neuroimaging: Design of a Multi-Modal EEG/fNIRS Instrument for Real-Time Use. Sensors.

[22] (2021). Core Specification. Bluetooth^®^ Technology Website. https://www.bluetooth.com/specifications/specs/core-specification-amended-5-2/.

[23] Andrzejak R.G., Lehnertz K., Mormann F., Rieke C., David P., Elger C.E. (2001). Indications of Nonlinear Deterministic and Finite-Dimensional Structures in Time Series of Brain Electrical Activity: Dependence on Recording Region and Brain State. Phys. Rev. E.

[24] Frey S., Spacone G., Cossettini A., Guermandi M., Schilk P., Benini L., Kartsch V. (2026). BioGAP-Ultra: A Modular Edge-AI Platform for Wearable Multimodal Biosignal Acquisition and Processing. IEEE Trans. Biomed. Circuits Syst..

[25] Hasan S., Pantha T., Arafat M.A. (2024). Design and Development of a Cost-Effective Portable IoT Enabled Multi-Channel Physiological Signal Monitoring System. Biomed. Eng. Adv..

