# Supplementary material for: Miniaturized Wearable System for Multimodal EEG/ECG/EMG Sensing and Real-Time Physiological Monitoring
Source: Micromachines (Basel). 2026 Jun 6;17(6):697. doi: 10.3390/mi17060697 (PMC13304197; doi:10.3390/mi17060697)
Supplement: Supplementary file 1 [file micromachines-17-00697-s001.zip › micromachines-4292387-supplementary.pdf]

Supporting Information for

Miniaturized Wearable System for Multimodal EEG/ECG/EMG Sensing and Real-Time Physiological Monitoring

Yunxiang Zhang [1,2,3], Xueyang Meng [1], Chengbang Lu [2,3], Yingning He [1,4,*] and Xiangyu Liang [2,3,*]

1 School of Physics and Optoelectronics, Xiangtan University, Xiangtan 411105, China; 202321521387@smail.xtu.edu.cn (Y.Z.); 202421521439@smail.xtu.edu.cn (X.M.)

2 Agricultural Genomics Institute at Shenzhen, Chinese Academy of Agricultural Sciences, Shenzhen 518120, China; luchengbang@caas.cn

3 Institute of Bast Fiber Crops, Center of Southern Economic Crops, Chinese Academy of Agricultural Sciences, Changsha 410205, China

4 State Key Laboratory of Molecular Engineering of Polymers, Fudan University, Shanghai 200438, China

* Correspondence: ynhe@xtu.edu.cn (Y.H.); liangxiangyu@caas.cn (X.L.)

S.I. Supporting results

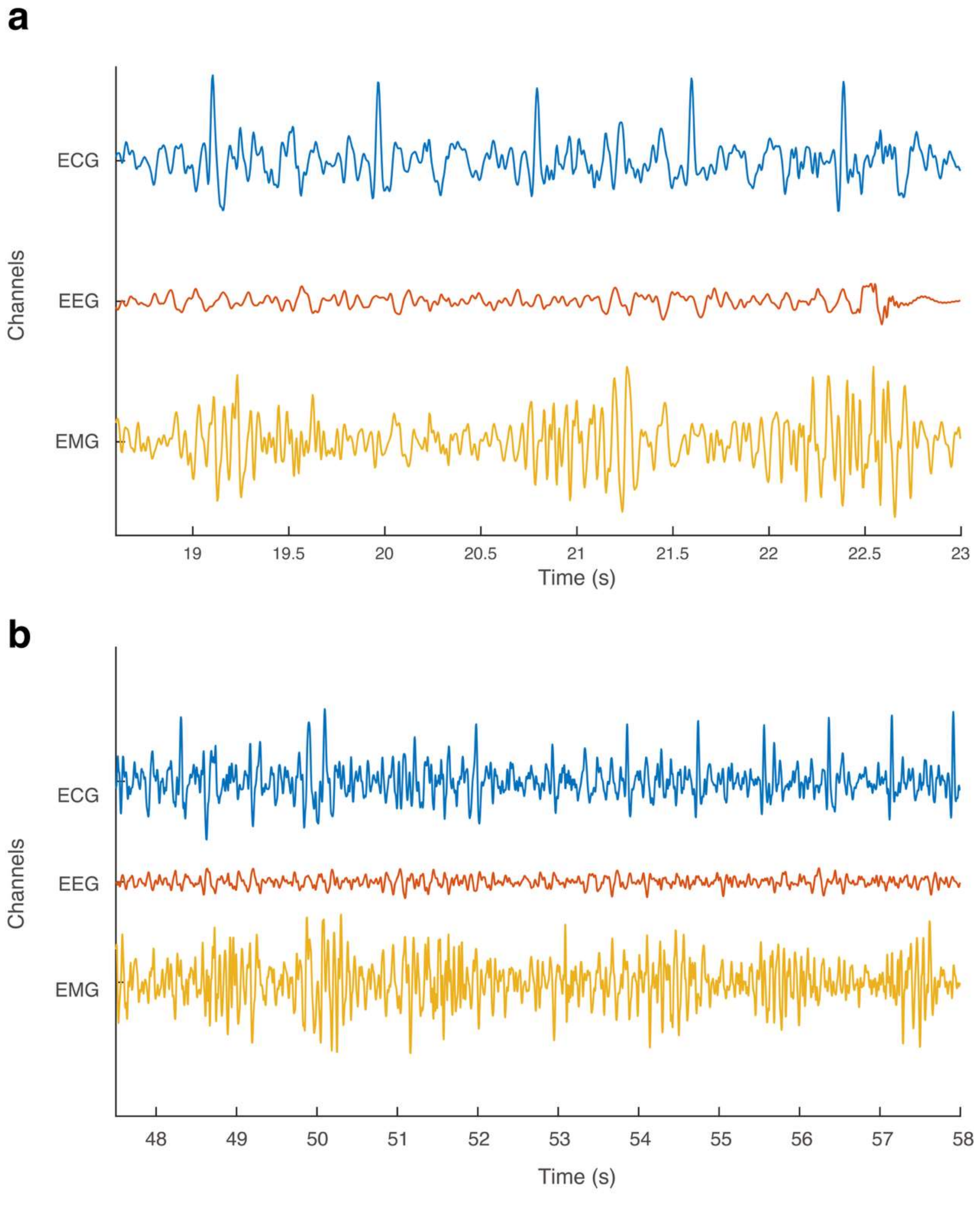

Figure S1. Representative simultaneous tri-modal recordings from two additional healthy subjects. (a, b) ECG, EEG, and EMG signals acquired in parallel from subjects S2 and S3, respectively, using the proposed wearable platform.

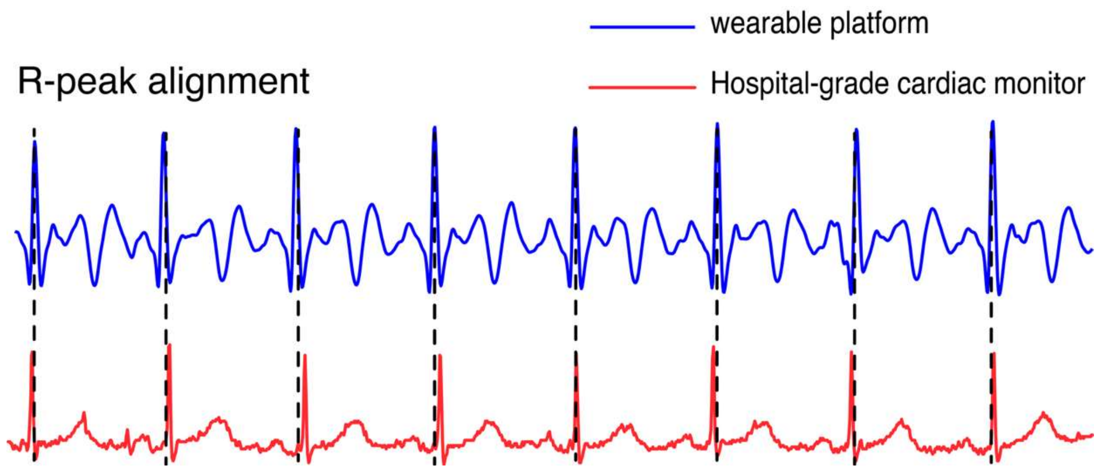

Figure S2. ECG validation against a clinical-grade ambulatory ECG recorder. Representative synchronized ECG waveforms show consistent R-peak alignment between the two systems.

Table S1. Bill of materials of the proposed wearable platform at single-unit prototype quantity.

Item	Specification	Qty	Unit Price (USD)	Subtotal (USD)
Analog front-end IC	ADS1299 (Texas Instruments)	1	44.18	44.18
Microcontroller	STM32F103C8T6 (STMicroelectronics)	1	1.21	1.21
BLE 5.2 module	RF-BM-BG22A3 (RF-star, EFR32BG22)	1	3.2	3.2
Power management ICs	LP5907MFX-3.3/NOPB; LM2664M6X/NOPB; TPS72325DBVR	3 types	various	2.79
LDO regulator (digital)	TLV70033DDCR (Texas Instruments)	1	0.39	0.39
Battery management IC	TP4056	1	0.63	0.63
Crystal oscillator	TAXM8M4RDBCCT2T, 8 MHz	1	0.56	0.56
Passive components	Resistors, capacitors, inductors, ferrite beads (assorted)	assorted	various	7.17
Connectors and headers	Electrode header, programming header, battery connector	assorted	various	3.78

Two-layer PCB fabrication	5.6 × 3.8 cm, 2-layer	1	2	2
Lithium-polymer battery	3.7 V	1	1.5	1.5
**Total**				**67.4**

Table S2. Quantitative agreement between the proposed platform and the clinical-grade ambulatory ECG recorder on synchronously recorded ECG.

Parameter	Subject	Proposed platform	Clinical-grade ECG recorder
Mean heart rate (bpm)	Subject 1	72.1	72
	Subject 2	76.8	77
Mean R-R interval (ms)	Subject 1	832	833
	Subject 2	782	779
QRS duration (ms)	Subject 1	83	82
	Subject 2	84	85
